# C-Reactive Protein in Arrhythmogenic Right Ventricular Dysplasia/Cardiomyopathy and Relationship with Ventricular Tachycardia

**DOI:** 10.4061/2010/919783

**Published:** 2010-08-24

**Authors:** Aimé Bonny, Nicolas Lellouche, Ivo Ditah, Françoise Hidden-Lucet, Martial T. Yitemben, Benjamin Granger, Fabrice Larrazet, Robert Frank, Guy Fontaine

**Affiliations:** ^1^Service de Cardiologie, Hôpital Saint Camille, 2 rue des pères camilliens, 94366 Bry-sur-Marne, France; ^2^Hôpital Henri-Mondor, Fédération de Cardiologie, 51, av Mal de Lattre de Tassigny, 94010 Créteil, France; ^3^Department of Internal Medicine, Wayne State University, 5475 Woodward Avenue, Detroit, MI 48202, USA; ^4^Unité de Rythmologie, Hôpital Pitié Salpêtrière, 47-83, Boulevard de l'Hôpital, 75651 Paris, Cedex 13, France; ^5^Service de Médecine, Centre Hospitalier Le Cateau, 28, Boulevard Paturle, 59360 Le Cateau- Cambresis, France; ^6^Département de Biostatistique, Hôpital Pitié Salpêtrière, 47-83, Boulevard de l'Hôpital, 75651 Paris, Cedex 13, France

## Abstract

*Background*. The relationship between C-reactive protein (CRP) elevation and ventricular tachycardia (VT) in arrhythmogenic right ventricular dysplasia/cardiomyopathy (ARVD/C) is unclear. *Methods and Results*. In 91 consecutive patients with either ARVD/C with or without VT (cases) or idiopathic right ventricular outflow tract (RVOT) tachycardia (controls), blood sampling were taken to determine CRP levels. In ARVD/C patients with VT, we analyzed the association between VT occurrences and CRP level. Sixty patients had ARVD/C, and 31 had idiopathic RVOT VT. Patients with ARVD/C had a significant higher level of CRP compared to those with RVOT VT (3.5 ± 4.9 versus 1.1 ± 1.2 mg/l, *P* = .0004). In ARVD/C group, 77%, (*n* = 46) patients experienced VT. Of these, 37% (*n* = 17) underwent blood testing for CRP within 24 h after the onset of VT and the remaining 63% (*n* = 29) after 24 h of VT reduction. CRP level was similar in ARVD/C patients with or without documented VT (3.6 ± 5.1 mg/l versus 3.1 ± 4.1 mg/l, *P* = .372). However, in patients with ARVD/C and documented VT, CRP was significantly higher when measured within 24 hours following VT in comparison to that level when measured after 24 h (4.9 ± 6.2 mg/l versus 3.0 ± 4.4 mg/l, *P* = .049). *Conclusion*. Inflammatory state is an active process in patients with ARVD/C. Moreover, there is a higher level of CRP in patients soon after ventricular tachycardia, and this probably tends to decrease after the event.

## 1. Introduction

Ventricular arrhythmias are a common finding with related poor outcomes in the setting of cardiomyopathies, either structural or electrical. Arrhythmogenic right ventricular dysplasia/cardiomyopathy (ARVD/C) is a primary heart muscle disease characterized by progressive atrophy of the right (but also left) ventricular myocardium with fibro-fatty replacement and the risk of electrical instability and sudden death [1–3]. The disease is often familial, and the pathophysiology is still unknown. Many studies have shown that ARVD/C implicates an inflammatory pathway with foci infiltrates with T lymphocytes cells in biopsy materials [4]. Whether inflammatory activity expressed by CRP concentration may play a role in pathogenesis of arrhythmias of ARVD/C has been little studied. 

Our study first evaluated the inflammatory activity expressed by CRP concentration in patients with ARVD/C. Secondly, we assessed the association between VT occurrence and CRP levels. Finally, we examined the temporal association between VT onset and CRP level (< or >24 hours). 

The study group was compared to patients with similar features of ventricular arrhythmias arising from right ventricle. 

## 2. Methods

### 2.1. Study Population

Enrollment took place from October 2001 to March 2008 in Hopital Pitie Salpitriere, Paris, France. The study population consisted of 105 consecutive patients. These patients were referred for ARVD/C screening based on right ventricular ectopic beats, right ventricular tachycardia, or family history of inherited arrhythmic disorder or sudden unexplained death. We reviewed their past medical histories and carried out a complete physical examination on all patients. All patients were subjected to cardiac tests (12-lead ECG, 24-hour ECG monitoring, signal averaged ECG, 2-D echocardiography, exercise ECG testing, coronary angiography, left and right ventricular contrast angiography and/or ventricular radionuclide imaging, and/or cardiac MRI) and blood sampled for CRP testing. The diagnosis of ARVD/C was according to the guidelines published by the Working Group of Myocardial and Pericardial Diseases of the European Society of Cardiology and the Scientific Council on Cardiomyopathies of the International Society and Federation of Cardiology [5]. This classification takes account of genetics, electrocardiographic depolarization and repolarization, and arrhythmic, structural, and histological factors. Based on this classification, the diagnosis of ARVD/C requires the presence of two major criteria, one major and two minor criteria, or four minor criteria. Right ventricular function is considered abnormal if right ventricular ejection fraction (RVEF) was less than 45%. Diagnosis of RVOT VT was based on standard guidelines [6]. Patients with suspected infection/inflammatory process during the screening were excluded from the study (*n* = 14). When CRP concentration was >10 mg/l, we particularly attempted to find any known cause. Indeed for each of these patients exhaustive diagnostic tests were performed to rule out a systemic infection (white cell account, urinary test, and chest X-ray) or other secondary causes of CRP elevation such as thrombosis or cancer. In patients with CRP >10 mg/l (4 patients), additional check up with body CT scan for tumor diagnosis or systemic inflammation was performed. We also excluded patients with recent (<1 week) electrical cardioversion, either external or by ICD. 

### 2.2. Ventricular Arrhythmia Diagnosis

**Figure 1:**
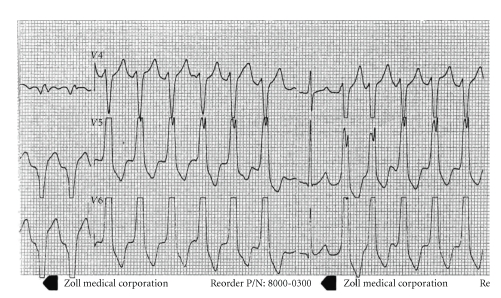
ECG of patient with ARVD/C and VT. A 32-year-old female with recurrent episodes of palpitation and near-syncope was admitted in ICU. Resting 12-lead ECG showed VT (wide complex tachycardia with capture) with rate of 150/min. SR was restored with bolus dose (600 mg during 1 min) of amiodarone. Blood sampling for CRP was drawn immediately after admission. Detailed examination led to the diagnosis of ARVD/C with family (brother) inheritance. ICU, Intensive care unit; VT, Ventricular tachycardia; CRP, C-reactive protein; SR, Sinus rhythm.

Premature ventricular contraction (PVC) was defined as wide ectopic beat with no anterograde P wave and sometimes retrograde P wave. More than 3 consecutive PVCs were classified as VT and sustained VT sought to last >30 sec. Wide complex tachycardia was differentiated between supraventricular tachycardia with conduction aberrancy and VT using Vereckei algorithm [7]. We diagnosed ventricular arrhythmia (VA) using resting 12-lead ECG (Figure 1), ambulatory holter monitoring, analysis of implantable cardioverter-defibrillator (ICD) storage electrogram, or analysis of automated external defibrillator (AED) during rescue. 

### 2.3. Blood Testing

On admission, before any invasive intervention, all patients underwent blood testing for C-reactive protein concentration using immunoturbidimetric Integra 400 Roche method. The time lapse between the last documented spontaneous VT event and blood sampling (VT/CRP < 24 h and VT/CRP > 24 h) was also recorded. We used only CRP measurements for characterization of inflammation. 

### 2.4. Statistical Analysis

Continuous variables are expressed as mean ± SD and statistical significance was assessed using the unpaired Student's t test or Mann-Whitney *U* test where applicable. Categorical variables, were summarized as proportion, and compared using the chi-square test or Fischer's exact test. The correlation between CRP levels and the rate of VT was assessed using Pearson correlation coefficient. Logistic regression was used to assess strength of association. In the multivariate model, only factors that attained statistical significance (*P* < .1) in the univariate analysis were included. The *χ* ^2^ test with a 2-tailed *P* value <.05 was considered statistically significant. Statistical analysis was performed with SPSS version 11.0.1 software (SPSS Inc).

## 3. Results

### 3.1. Characteristics of the Baseline Population

**Figure 2:**
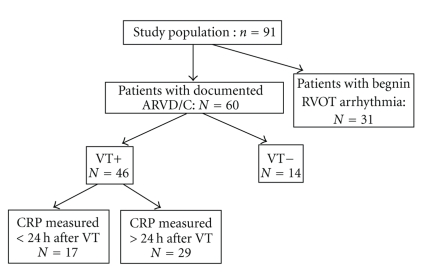
Flowchart of study population. Study population included case group (ARVD/C) and control (RVOT ). Among 60 ARVD/C patients, 46 had VT of whom 17 underwent CRP testing less than 24 h after VT occurrence. ARVD/C, Arrhythmogenic right ventricular tachycardia; RVOT, Right ventricular outflow tract; CRP, C-reactive protein; VT, Ventricular tachycardia.

Our study sample included 91 patients. The baseline characteristics of our population are shown in Table 1. The mean age of the study population was 39 ± 13 years with 73% being male. The prevalence of cardiovascular risk factors was low: 4% hypertension, 14% smokers, 8% hypercholesterolemia, and none were diabetic. Figure 2 shows the distribution of the study population by ARVD/C, VT occurrence, and CRP measurement timing from VT occurrence. 

### 3.2. Comparison of Patients with ARVD/C and RVOT Tachycardia

**Figure 3:**
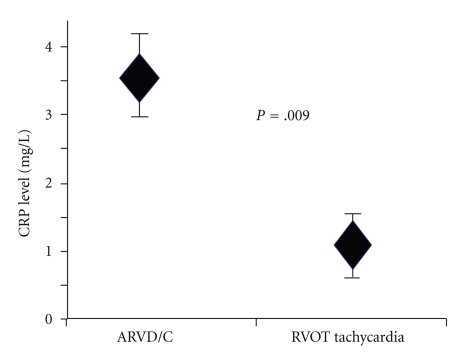
CRP levels of patients ARVD/C and RVOT tachycardia. Comparison of CRP concentrations between patients with ARVD/C (case) and RVOT tachycardia (control). CRP, C-reactive protein; ARVD/C, Arrhythmogenic right ventricular dysplasia/tachycardia; RVOT, Right ventricular outflow tract.

Sixty patients had ARVD/C, and 31 patients had RVOT tachycardia. Their characteristics are shown on Table 1. Study populations were similar in age distribution, history of hypertension, dyslipidemia, smoking, statin and aspirin use. However patients with ARVD were more likely to be male (85% versus 48%, *P* < .05) and have right ventricular dysfunction (23% versus 0%, *P* = .004) to patients to RVOT tachycardia. While all patients in the control group experienced VT, only forty six (77%) ARVD/C patients experienced it (*P* = .037). Nineteen (32%) ARVD/C patients had ICD before inclusion whereas none of the RVOT patients had ICDs. Finally as shown in Figure 3, the mean CRP level was 3.5 ± 4.9 mg/l in ARVD/C group and 1.1 ± 1.2 mg/l in the control group (*P* = .009).

Table 2 shows the multivariate analysis. Only the male sex, CRP level and spontaneous VT were independently associated with ARVD/C.

### 3.3. Ventricular Arrhythmia Features of Overall Population

The characteristics of VA of our study population are shown in Table 3. There was no correlation between VT rate and CRP levels (*r* = 0.09; *P* = .542). CRP levels were similar in patients with or without clinical manifestations (syncope or aborted sudden cardiac death) due to VA (*P* = .886).

### 3.4. Comparison between ARVD/C Patients with and without VT

In the ARVD/C group, 46 (77%) patients had at least one documented spontaneous episode of VT (>3 consecutives PVC). The comparison between ARVD/C with and without VT is shown in Table 4. The occurrence of VT was not related to structural changes in the right ventricle. Indeed, RV dysfunction (RVEF < 40%) assessing by imaging techniques (2-D echocardiogram, radionuclide ventriculography, cardiac MRI or ventricular angiography) was similar in patients with or without VT (24% versus 21% resp., *P* = .99). CRP concentrations did not differ significantly with respect to previous history of VT (VT+ versus VT−). The mean CRP levels in the VT and no-VT subgroups were 3.6 ± 5.1 and 3.1 ± 4.1 mg/l (*P* = .372), respectively.

### 3.5. Comparison of ARVD/C Patients with VT According to CRP Timing Measurement

**Figure 4:**
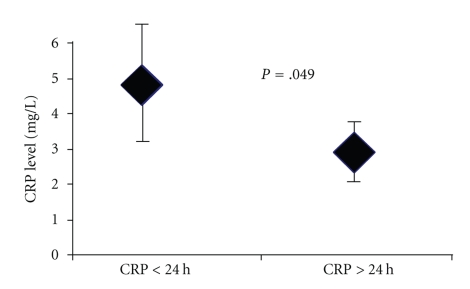
CRP level in patients with ARVD/C and VT according to the timing between CRP measurement and VT occurrence (< or >24 h). CRP concentration of ARVD/C patient with the history of VT was significantly greater when blood work-up for CRP was drawn within 24 h of VT onset compared to the time lapse >24 h after VT restoration of SR. CRP, C-reactive protein; ARVD/C, Arrhythmogenic right ventricular dysplasia/cardiomyopathy; VT, Ventricular tachycardia; SR, Sinus rhythm.

Of 46 ARVD/C patients with VT, blood testing for CRP measurement was performed within 24 h after VT occurrence (CRP/VT < 24 h) in 17 (37%) patients. Both subgroups were similar regarding cardiovascular risk factors, right ventricular contractility and ventricular rate during VT (Table 5). As shown in Figure 4, CRP levels were significantly higher when measured in less than 24 h after VT occurrence (4.9 ± 6.2 mg/l versus 3.0 ± 4.4 mg/l, *P* = .049).

## 4. Discussion

### 4.1. Major Findings

This study shows that ARVD/C is associated with inflammation, as indicated by higher CRP concentration. The inflammatory state is similar in all ARVD/C patients, regardless of the history of ventricular tachyarrhythmia. CRP levels were significantly higher when blood sampling was performed in the first 24 hours of VT manifestation. 

### 4.2. Inflammation and Cardiovascular Outcomes

C-reactive protein is a biomarker of inflammation and its increase level predicts cardiovascular events such as stroke, coronary heart disease, or peripheral vascular disease [8, 9]. Serum CRP level greater than 2 mg/l has been shown to predict theses cardiovascular events [10] and anti-inflammatory agents such as statins have demonstrated a reduction of cardiovascular mortality in patients with normal lipid profile [11]. 

It is known that systemic inflammation is associated with arrhythmia occurrence. This association has clearly been established during atrial fibrillation (AF). It has been shown that increased CRP levels are associated with greater risk of AF recurrence after electrical cardioversion [12]. Moreover, dilated cardiomyopathy patients with AF have higher inflammation activation than those without AF [13]. 

Ventricular arrhythmia (VA) occurrence is associated with significantly elevated proinflammatory markers such as IL-6 and highly sensitive C-reactive protein (hs-CRP) in implantable cardioverter-defibrillator (ICD) patients with structural heart disease [14]. Additionally, during acute myocardial ischemia, patients with malignant VA experience higher systemic inflammation activation than those without VA [15].

Myocardial inflammation seems to play an important role in chronic heart failure and cardiomyopathies. Despite all reports, a clear correlation with clinical outcome and survival rates has not been documented and clinical studies have not shown consistent beneficial effects of an anti-inflammatory therapy [16–18].

Enhanced inflammatory response is related to the development of ventricular tachycardia or ventricular fibrillation (VT/VF) after ST-elevation myocardial infarction (STEMI) [19]. Consistent with the later, statins which have anti-inflammatory properties [20] are associated with decreased incidence of ventricular tachycardia (VT) [21–24]. However, recent data has suggested that inflammatory biomarkers such as IL-6, TNF-alpha, hsCRP, and BNP are not predictive of intermediate-term risk of ventricular tachyarrhythmias in stable chronic heart failure [25]. 

Finally, whether the activation of systemic inflammation is the cause or the consequence of ventricular arrhythmias is unknown. 

### 4.3. Inflammation during ARVD/C and Its Relationship with VT Occurrence

As demonstrated in previous studies, the risk profile which emerges from retrospective analysis of clinical and pathologic series, including fatal cases, is characterised by young age, competitive sport activity, malignant family background, extensive right ventricular disease with low ejection fraction and left ventricular involvement, syncope, and episodes of complex ventricular arrhythmias [3, 26].

Previous studies have found foci infiltrates with T lymphocytes cells in biopsy materials of patients with ARVD/C [27–32]. Therefore, ARVD/C may be considered as an appropriate model to study the relationship between inflammation and ventricular arrhythmia which is a major clinical feature of this inherited arrhythmogenic disease. Consistent with this, we found that the baseline CRP level was significantly higher in patients with ARVD/C compared to RVOT tachycardia patients. We assumed that it would be worthy to compare these two groups with age and sex match controls. Nevertheless, we hypothesized that healthy people should have lower inflammatory state rather than both studied groups. 

Ventricular tachycardia is a common clinical feature of ARVD/C, no matter of right ventricular contractility changes evaluating by RVEF as shown our results. This finding supports the already known fact that the early “concealed” phase is characterized by a propensity toward ventricular tachyarrhythmia and sudden cardiac death (SCD) in the setting of well-preserved morphology, histology, and ventricular function [33]. 

The question of whether the elevation of this inflammatory biomarker is the cause or consequence of ventricular arrhythmia manifestation in the setting of ARVD/C is a major concern. Our study aimed at determining if VA causes the elevation of CRP in the setting of ARVD/C. For this purpose, we analysed all ambulatory or inpatient 12-leads ECG, 24-h ECG monitoring and storage and ICD electrograms of patients at the admission. CRP testing was drawn at the admission prior to any invasive intervention such as EP study or ICD implantation. Therefore, neither programmed ventricular pacing nor ICD therapy could influence levels of inflammatory state of this sample.

We found that CRP level was significantly higher when measured immediately after VT occurrence (<24 h). However our retrospective cross-sectional study design was not appropriate to establish if this association was a cause or a consequence. The results suggest that the CRP level may not be associated with aetiology or pathogenesis of ventricular tachycardia. Activation of systemic inflammation seems to be the consequence rather than the cause of ventricular arrhythmia in these patients. 

Our findings support the hypothesis that there is a higher level of CRP in ARVD/C patients soon after ventricular tachycardia and that this probably tends to decrease after the ventricular tachycardia. However the cause of the elevation of CRP in these patients and its relation to ventricular tachycardia are unclear.

### 4.4. Study Limitations

First, we did not use the nephelometric technique in assessing highly sensitive C-reactive protein (hs-CRP) which is about a 10-fold more sensitive than the immunoturbidimetric assay used in this trial. That was due to the fact that nephelometric technique was not available in our centre at the beginning of inclusion. Therefore, our results are less sensitive. Second, this is a retrospective observational study with some deficiencies in rigorous establishment of serial timing between blood testing for CRP concentration and the occurrence of arrhythmic events. Third, CRP levels have been analyzed in the plasma only and no correlation with myocardial inflammation is given as no biopsies have been taken. Accordingly, conclusions are drawn from the plasma into the myocardium. Fourth, a true control group represented by healthy people matched by age and sex is lacking. Fifth, our control group could not diagnose ARVD/C at the moment of the study, but the diagnosis of idiopathic right ventricular outflow tract seems to be an exclusion diagnosis with the possible disease progression to ascertaine ARVD/C.

## 5. Conclusion

This study is the first to demonstrate that systemic inflammation expressed by high C-reactive protein concentration is a more active process soon after spontaneous ventricular arrhythmia in ARVD/C patients, independently of ventricular rate. 

##  Conflict of Interest

The authors declared no conflict of interest.

## Figures and Tables

**Table 1:** Baseline characteristics of patients with ARVD/C and RVOT tachycardia.

	ARVD/C (*n* = 60) Mean ± SD	RVOT tachycardia (*n* = 31) Mean ± SD	*P* value
Age (years)	39 ± 14	38 ± 12	.631
Male (%)	85	48	**.0002**
Hypertension (%)	8	0	.123
Hypercholesterolemia (%)	4	12	.209
Smokers (%)	17	19	.871
RV dysfunction (%)	23	0	**.004**
Statin (%)	5	0	.206
Aspirin (%)	3	3	.978
Documented VT (%)	77	100	**.037**
VT-R	195 ± 46	182 ± 41	.374
ICD	38	10	.006
CRP measured <24 h following VT (%)	37	34	.864

ARVD/C, Arrhythmogenic right ventricular dysplasia/cardiomyopathy; RVOT, Right ventricular outflow tract; RV, Right ventricular; VT, Ventricular tachycardia; VT-R, Ventricular tachycardia rate; ICD, Implantable cardioverter-defibrillator; CRP, C-reactive protein.

**Table 2:** Multivariate analysis* in patients with ARVD/C or RVOT tachycardia.

	*P* value	95% Confidence interval
Male (%)	**.024**	1.19–11.85
RV dysfunction (%)	.977	0.005–0.008
Documented VT (%)	.718	0.24–2.67
ICD	.712	0.26–6.97
CRP concentration	**.025**	1.07–2.78

ARVD/C, Arrhythmogenic right ventricular dysplasia/cardiomyopathy; RVOT, Right ventricular outflow tract; RV, Right ventricular; VT, Ventricular tachycardia; VT-R, Ventricular tachycardia rate; ICD, Implantable cardioverter-defibrillator; CRP, C-reactive protein.

**Table 3:** Characteristics of ventricular arrhythmias of overall population.

	Diagnostic tools			Heart rate		
R. ECG	E. testing	A. ECG	ICD/AED	ARVD/C	RVOT	*P*
42 (63%)	7 (10%)	7 (10%)	13 (19%)	195 ± 46	182 ± 41	.374

ARVD/C, Arrhythmogenic right ventricular dysplasia/cardiomyopathy; RVOT, Right ventricular outflow tract; RV, Right ventricular; VT, Ventricular tachycardia; VT-R, Ventricular tachycardia rate; ICD, Implantable cardioverter-defibrillator; CRP, C-reactive protein.

**Table 4:** Baseline characteristics of ARVD/C patients with or without documented VT.

	VT+ (*n* = 46) Mean ± SD	VT− (*n* = 14) Mean ± SD	*P* value
Age (years)	40 ± 15	38 ± 8	.641
Male (%)	87	79	.423
Hypertension (%)	11	0	.561
Hypercholesterolemia (%)	16	0	.313
Smokers (%)	14	27	.367
RV dysfunction (%)	24	21	.998
Statin (%)	7	0	.998
Aspirin (%)	4	0	.998
CRP level (mg/L)	3.6 ± 5.1	3.1 ± 4.1	.372

ARVD/C, Arrhythmogenic right ventricular dysplasia/cardiomyopathy; RVOT, Right ventricular outflow tract; RV, Right ventricular; VT, Ventricular tachycardia; VT-R, Ventricular tachycardia rate; ICD, Implantable cardioverter-defibrillator; CRP, C-reactive protein.

**Table 5:** Baseline characteristics of ARVD/C and documented VT patients with or without CRP level measured within the 24 hours following VT.

	CRP < 24 h (*n* = 17) Mean ± SD	CRP > 24 h (*n* = 29) Mean ± SD	*P* value
Age (years)	44 ± 18	37 ± 13	.196
Male (%)	88	86	.998
Hypertension (%)	20	45	.283
Hypercholesterolemia (%)	27	9	.198
Smokers (%)	20	10	.630
RV dysfunction (%)	31	21	.698
Statin (%)	12	3	.285
Aspirin (%)	6	3	.998
VT-R	188 ± 41/min	201 ± 51/min	.552

ARVD/C, Arrhythmogenic right ventricular dysplasia/cardiomyopathy; RVOT, Right ventricular outflow tract; RV, Right ventricular; VT, Ventricular tachycardia; VT-R, Ventricular tachycardia rate; ICD, Implantable cardioverter-defibrillator; CRP, C-reactive protein.
